# Genome Sequence of *Fusarium graminearum* ITEM 124 (ATCC 56091), a Mycotoxigenic Plant Pathogen

**DOI:** 10.1128/genomeA.01209-17

**Published:** 2017-11-09

**Authors:** Antonio Zapparata, Daniele Da Lio, Stefania Somma, Isabel Vicente Muñoz, Luca Malfatti, Giovanni Vannacci, Antonio Moretti, Riccardo Baroncelli, Sabrina Sarrocco

**Affiliations:** a Department of Agriculture, Food and Environment, University of Pisa, Pisa, Italy; b Institute of Sciences of Food Production, National Research Council, Bari, Italy

## Abstract

*Fusarium graminearum* is among the main causal agents of *Fusarium* head blight (FHB), or scab, of wheat and other cereals, caused by a complex of *Fusarium* species, worldwide. Besides causing economic losses in terms of crop yield and quality, *F. graminearum* poses a severe threat to animal and human health. Here, we present the first draft whole-genome sequence of the mycotoxigenic *Fusarium graminearum* strain ITEM 124, also providing useful information for comparative genomics studies.

## GENOME ANNOUNCEMENT

*Fusarium* head blight (FHB) of wheat is a major disease worldwide, with *Fusarium graminearum* being the main causal agent of the species complex (1). Notably, *F. graminearum* was recently ranked fourth in a top 10 fungal plant pathogen list (2). FHB not only directly affects the grain production but also poses a severe threat to plant, animal, and human health due to the accumulation of mycotoxins on kernels and wheat products.

*F. graminearum* ITEM 124 (http://server.ispa.cnr.it/ITEM/Collection/), also known as ATCC 56091, was isolated in 1976 from rice harvested in Vercelli, Piemonte, Italy. *F. graminearum* ITEM 124 is able to produce deoxynivalenol (DON), the best-known mycotoxin belonging to trichothecenes (3). This class of mycotoxins acts as an inhibitor of cell protein synthesis (4). The ability of *F. graminearum* ITEM 124 to produce DON on wheat and rice kernels has been assessed previously (5, 6). Molecular chemotype detection, performed according to Somma et al. (7), identified the strain *F. graminearum* ITEM 124 as chemotype 15-acetyl DON (15ADON). Chemical analyses confirmed the production of DON by ultraperformance liquid chromatography (UPLC) with a photodiode array (PDA) assay (8).

A competition test on rice kernels inoculated with *F. graminearum* ITEM 124 and the beneficial fungus *Trichoderma gamsii* T6085 highlighted a reduction of the amount of DON to almost 92% as a direct consequence of a decreased biomass of the pathogen. Moreover, the growth of *F. graminearum* ITEM 124 has been reduced by the presence of *T. gamsii* T6085 in dual confrontation assays. *F. graminearum* ITEM 124 is overgrown by *T. gamsii* T6085 under *in vitro* conditions, and the latter produces short loops and coilings on pathogen hyphae, which are typical mycoparasitic traits (5, 6, 9).

The genome of *F. graminearum* ITEM 124 was sequenced using Illumina paired-end sequencing technology by BMR Genomics (Padua, Italy). Paired reads of 300 bp (3.16 Gbp; average coverage, 38×) were assembled using SPAdes v3.8.2 (10). The nuclear genome of *F. graminearum* ITEM 124 consists of 67 sequence scaffolds with a total assembly length of 36.88 Mbp (*N*_50_, 1,518,396; *L*_50_, 6), 48.18% GC content, and a maximum scaffold size of 7,305,194 bp. The completeness of the assembly was assessed using BUSCO v12 (11), which estimated the genome sequence to be 99.86% complete. The nuclear genome was annotated using the MAKER2 pipeline (12). Overall, 11,827 protein-coding gene models were predicted. Analysis with SignalP 4.1 (13) revealed that 1,288 predicted proteins (10.9% of the proteome) contain a secretion signal peptide.

The genome sequence of the mycotoxigenic *F. graminearum* ITEM 124 provides a novel source for comparative genomics studies and also represents a useful platform to study fungal-fungal interactions, such as the mycoparasitic relationship established with the beneficial isolate *T. gamsii* T6085, whose genome is also available (14).

### Accession number(s).

This whole-genome shotgun project has been deposited in GenBank under accession number NQOC00000000 (BioProject PRJNA397367; BioSample SAMN07455307). The version described in this paper is NQOC01000000.
